# *Chlamydia pneumoniae* Seropositivity and Acute Coronary Syndromes: A Case–Control Study of the Infectious–Inflammatory Axis

**DOI:** 10.3390/medicina62061107

**Published:** 2026-06-06

**Authors:** Lujain Fouad Khalaf, Rozan Fouad Khalaf, Shady Salah Bagady, Romaysaa Fouad Khalaf, Rodina Amro Amin, Abdalla O. Manaa, Mohamad Salah Bagady, Ahmad Aljada

**Affiliations:** 1 College of Medicine, Alfaisal University, Riyadh 11533, Saudi Arabia; lfkhalaf@alfaisal.edu (L.F.K.); sbagady@alfaisal.edu (S.S.B.); ramin@alfaisal.edu (R.A.A.); mbagady01@alfaisal.edu (M.S.B.); 2 College of Medicine, Alexandria University, Alexandria 26571, Egypt; rozan.fouad2101@alexmed.edu.eg (R.F.K.); abdallah.fadal1601@alexmed.edu.eg (A.O.M.); 3 Department of Biochemistry and Molecular Medicine, College of Medicine, Alfaisal University, Riyadh 11533, Saudi Arabia

**Keywords:** acute coronary syndromes, *Chlamydia pneumoniae*, atherosclerosis, C-reactive protein, case–control study

## Abstract

*Background and Objectives:* Classical cardiovascular risk factors account for only a fraction of acute coronary syndromes (ACSs), and chronic *Chlamydia pneumoniae* infection has been proposed as a contributor to atherogenesis through persistent inflammation and endothelial dysfunction. We tested whether *C. pneumoniae* infection is independently associated with ACS by quantifying seroprevalence, inflammatory markers, and their relationship with conventional cardiovascular risk factors. *Materials and Methods:* In a prospective case–control design, we enrolled 47 patients with ACSs (29 with acute myocardial infarction and 18 with unstable angina) and 53 age- and locality-matched controls at Alexandria University Hospital. The clinical evaluation comprised electrocardiography, echocardiography, lipid profile, and high-sensitivity C-reactive protein (CRP). *C. pneumoniae*-specific IgG and IgM were measured by ELISA, with positive samples confirmed by microimmunofluorescence. Logistic regression models were adjusted for age, sex, hypertension, diabetes, dyslipidemia, and smoking. *Results:* IgM was undetectable in all 100 participants, excluding acute infection. IgG seropositivity was higher in cases than in controls (83.0% vs. 60.4%; OR: 3.20; 95% CI: 1.23–8.30; *p* = 0.017) and remained suggestive after multivariable adjustment (adjusted OR: 4.59; 95% CI: 1.33–18.28; *p* = 0.021), although the estimate is imprecise and does not meet our prespecified multivariate threshold of *p* < 0.01. Within the ACS cohort, IgG seropositivity was not significantly associated with CRP elevation (Fisher’s exact *p* = 1.000). CRP elevation was near-universal in cases (93.6%) and absent in controls (0%; *p* < 0.001). *Conclusions:* Chronic *C. pneumoniae* infection was associated with ACS in unadjusted analysis, with a suggestive but underpowered signal after multivariable adjustment, although the observational design precludes causal inference, and reverse causality cannot be excluded. Prospective studies using direct pathogen detection are required to determine whether the association reflects a contributory mechanism or shared susceptibility.

## 1. Introduction

Acute coronary syndromes (ACSs) include ST-elevation myocardial infarction, non-ST-elevation myocardial infarction, and unstable angina. Together, they account for a substantial share of global cardiovascular mortality [1,2]. Contemporary epidemiological data demonstrate substantially elevated mortality rates among male populations and socioeconomically disadvantaged regions [3]. The clinical and economic burden of ACS continues to grow worldwide [4], as adoption of Western dietary patterns and sedentary lifestyles drives rising incidence in developing countries [5].

Risk stratification in ACSs is imperfect. Between 80% and 90% of patients with coronary heart disease harbor at least one conventional risk factor [6], yet patients lacking any documented traditional risk factor still make up roughly 10.5% of non-ST-segment elevation myocardial infarction cohorts [7]. Several studies have reported worse outcomes, including higher mortality, among ACS patients without standard modifiable risk factors (SMuRFs) than among those with recognized SMuRFs [8,9,10]. A large international cohort analysis found no protective mortality benefit from SMuRF absence after multivariate adjustment [10]. Adding novel biomarkers to traditional risk factors may improve prognostic accuracy and refine treatment in high-risk ACS [11].

Atherosclerosis is now recognized as a chronic inflammatory process, with inflammation contributing to disease initiation, plaque progression, and acute plaque complications [12,13]. CRP independently predicts cardiovascular events, with prognostic weight that exceeds the traditional risk factor set [14,15]. The liver produces CRP largely in response to interleukin-6, and CRP has held up as a prognostic marker across both primary and secondary prevention cohorts [16,17]. Soluble CD40 ligand, adiponectin, and matrix metalloproteinase-9 add incremental discrimination [14]. Experimental data point to a direct contribution to atherogenesis: CRP may signal through pro-inflammatory pathways rather than passively marking inflammation [15]. Interest in anti-inflammatory therapy and in refined risk stratification has grown as a result [18].

Chronic infection is another variable under scrutiny. *Chlamydia pneumoniae*, cytomegalovirus, and *Helicobacter pylori* have each been recovered from atherosclerotic plaque, and each is linked epidemiologically to elevated cardiovascular risk [19,20]. Vascular injury from these organisms can be direct, through endothelial infection, or indirect, via cytokine release and acute-phase protein induction [21,22]. Beyond any single organism, the “infectious burden” hypothesis weighs cumulative exposure to multiple chronic pathogens as the relevant driver of atherosclerotic progression [23,24]. Pathways most often invoked are endothelial dysfunction, foam cell transformation, and persistent inflammatory mediator production [25]. Causal proof in human populations is lacking. Large-scale antibiotic intervention trials failed to show clinical benefit [26].

*C. pneumoniae* seropositivity has been associated with angiographically confirmed coronary artery disease in several populations [27,28]. After adjustment for conventional cardiovascular risk factors, odds ratios for the association between chronic *C. pneumoniae* infection and coronary heart disease fall between 2.3 and 2.9 [27]. Seroepidemiological data, immunohistochemistry of atherosclerotic specimens, and in vitro mechanistic work all point toward *C. pneumoniae* involvement in atherogenesis [29]. *C. pneumoniae* antigens concentrate within atherosclerotic lesions across the arterial tree but are virtually absent from histologically normal arterial segments [30].

Causality remains disputed. Some investigations reported strong associations [29,31]. Methodological shortcomings and poor reproducibility across cohorts, however, have fueled doubt [32,33]. Investigators have recovered *C. pneumoniae* DNA and antigens from atherosclerotic plaques [34,35]. In animal models, experimental *C. pneumoniae* inoculation accelerates atherosclerosis [31]. Koch’s postulates for microbial causation remain incompletely fulfilled [36]. At the molecular level, *C. pneumoniae* appears to modulate atheroma biology through inflammatory signaling and dysregulated lipid metabolism [34,35]. Early small-scale antibiotic trials raised hopes of therapeutic benefit. Large randomized controlled trials in stable coronary artery disease populations, however, showed no clinical efficacy [34]. Meta-analyses of prospective cohort data indicate modest associations, strongest among younger patients, though effect sizes attenuate after rigorous confounder adjustment [37]. *C. pneumoniae* likely acts as a facilitator of atherosclerotic pathogenesis rather than a direct cause [35,38].

When persistent *C. pneumoniae* infection coincides with systemic inflammation, reflected by elevated CRP, coronary risk may rise substantially [39]. Not all data agree. Other studies found no significant association between *C. pneumoniae* seropositivity and inflammatory biomarkers or adverse outcomes in ACS cohorts [40]. The interaction between infection and inflammation in atherosclerotic cardiovascular disease is not settled.

Traditional risk factors alone do not account for all coronary artery disease. The potential contribution of infectious agents such as *C. pneumoniae* to ACS warrants investigation. One critical methodological constraint deserves emphasis: serological assessment of past exposure cannot distinguish prior immunological contact from active pathogenic involvement in the vascular wall. Clarifying how infection and inflammation interact in atherogenesis, with direct pathogen detection methods such as PCR, could inform future risk stratification and therapeutic approaches in high-risk populations.

## 2. Materials and Methods

### 2.1. Ethical Considerations

This study was reviewed and approved by the Institutional Review Board of the Faculty of Medicine, Alexandria University. We followed the 1964 Declaration of Helsinki, as amended, and the standards of the institutional research committee. Written informed consent was obtained from every participant prior to any study procedure. Withdrawal was available at any point and without consequence to ongoing medical care.

### 2.2. Study Population

Recruitment took place in the Cardiology and Angiology Department at Alexandria University Hospital, Egypt, between May 2025 and March 2026, under a prospective case–control design. Forty-seven consecutive patients with ACS and 53 age- and locality-matched healthy controls were enrolled. Sample size was estimated a priori from a comparison of two independent proportions (two-sided test; α = 0.05; 80% power). At least 42 participants per group were required to detect a 30% absolute difference in *C. pneumoniae* seropositivity. Our 30% threshold reflected seroprevalence differences from early case–control work on *C. pneumoniae* and coronary artery disease [27,28]. The observed difference was 22.6% (83.0% vs. 60.4%), below this threshold. Power for the primary serological comparison was therefore marginal. The calculation used an independent-groups framework rather than matched methods, so the power estimate is approximate.

Cases were aged 32–75 years and presented within 24 h of symptom onset. ACS was diagnosed by universal definition criteria. Acute myocardial infarction required at least two of the following: characteristic chest pain > 20 min, evolutionary electrocardiographic changes (ST-segment elevation or depression ≥ 1 mm in ≥2 contiguous leads, or new pathological Q waves), and cardiac biomarker elevation (CK-MB > 25 ng/mL or LDH1:LDH2 ratio > 1.0). Unstable angina was new-onset severe angina, accelerating angina, or rest angina without biomarker elevation. Controls had no cardiovascular history, normal electrocardiograms, and no cardiovascular symptoms. All were drawn from the same geographic catchment as cases. The exclusion criteria were previous myocardial infarction, active inflammatory disease, malignancy, immunosuppressive therapy, chronic renal failure (creatinine > 2.0 mg/dL), autoimmune disease, or surgery within the prior 30 days.

### 2.3. Clinical Assessment

Trained personnel, blinded to serological results, collected standardized clinical data on structured case report forms. Definitions of cardiovascular risk factors followed current guideline criteria. A participant was considered hypertensive at systolic blood pressure ≥ 140 mmHg or diastolic ≥ 90 mmHg (mean of three readings), or while taking antihypertensive medication. The threshold for diabetes was fasting plasma glucose ≥ 126 mg/dL or current antidiabetic therapy. Dyslipidemia required at least one of the following: total cholesterol > 200 mg/dL, LDL ≥ 130 mg/dL, HDL < 40 mg/dL in men or <45 mg/dL in women, triglycerides > 180 mg/dL, or use of lipid-lowering therapy. Smoking history was recorded as never, former (cessation > 30 days), or current. Cumulative exposure was captured by the smoking index, calculated as cigarettes per day multiplied by years smoked. All participants underwent twelve-lead electrocardiography and transthoracic echocardiography. Cardiac biomarkers were sampled at admission and at 6, 12, and 24 h by automated immunoassay, against reference ranges of CK-MB 0–25 ng/mL, total CK 30–200 U/L (men) and 20–180 U/L (women), and LDH 140–280 U/L. The left ventricular ejection fraction was measured by biplane Simpson’s method, with values < 50% classified as abnormal.

### 2.4. Laboratory Analyses

Venous blood was drawn within 12 h of admission and centrifuged at 3000× *g* for 15 min at 4 °C, and serum aliquots were stored at −80 °C until batch analysis. CRP was measured by a high-sensitivity latex-enhanced immunoturbidimetric assay (HUMATEX CRP; HUMAN Diagnostics, Wiesbaden, Germany) (Hitachi 917; Roche Diagnostics, Mannheim, Germany), with an analytical sensitivity of 0.1 mg/L and a coefficient of variation under 5%. Values of CRP ≥ 6 mg/L were treated as elevated, in line with published cardiovascular risk stratification cut-off points. Lipid panels were run on a Hitachi 917 analyzer using enzymatic colorimetric methods, and LDL cholesterol was calculated by the Friedewald equation when triglycerides were below 400 mg/dL.

### 2.5. Chlamydia pneumoniae Serology

*C. pneumoniae*-specific IgG and IgM were detected by commercial enzyme-linked immunosorbent assay (ELISA, VIRCELL SL, Granada, Spain), with positive samples confirmed by microimmunofluorescence. All serological work was performed by laboratory personnel blinded to clinical status. Sera were diluted 1:100 and processed per the manufacturer’s protocol, with incubation at 37 °C ± 1 °C. Each plate carried duplicate samples alongside positive, negative, and cut-off controls. The antibody index was (sample optical density/cut-off control mean optical density) × 10. IgM positivity was defined as index ≥ 11. Index ≤ 9 was negative. Intermediate values (9.1–10.9) were excluded. IgG seropositivity was defined by validated cut-off points using the same approach. All samples were run in duplicate with a coefficient of variation below 10%. Discordant results were repeated. Quality control relied on external proficiency testing programs. Serology has a recognized limit. Antibody detection signals immunological exposure but cannot confirm active vascular infection or separate past from ongoing pathogenic involvement.

### 2.6. Statistical Analysis

Analyses were performed in SPSS 23.0 (IBM Corp., Armonk, NY, USA), with figures generated in GraphPad Prism 8.0 (GraphPad Software, San Diego, CA, USA). Continuous variables, expressed as means ± SD, were compared by Student’s *t*-test under normality (assessed by Shapiro–Wilk) and by the Mann–Whitney U test otherwise. For categorical variables, Pearson’s chi-square test was used, with Fisher’s exact test substituted whenever an expected cell count fell below 5. Odds ratios (ORs) with 95% confidence intervals (CIs) for the link between *C. pneumoniae* seropositivity and ACS came from logistic regression. The confounders specified a priori were age, sex, smoking, hypertension, diabetes, and dyslipidemia. We then fitted unconditional multivariate logistic regression models for the independent associations. Conditional logistic regression, accounting for the matched-pairs structure, was not used. Implications of this analytic choice are addressed in our limitations discussion. Age and locality served as matching variables. Sex was not used as a matching variable. It entered the models as a covariate. We applied a significance threshold of *p* < 0.05 for bivariate comparisons and *p* < 0.01 for multivariate analyses as a partial adjustment for multiple testing. The bivariate threshold does not formally control the false discovery rate. All tests were two-tailed.

## 3. Results

### 3.1. Study Population and Baseline Characteristics

A total of 100 participants were enrolled in this case–control study, comprising 47 patients with acute coronary syndromes (ACSs) and 53 healthy controls matched for age and locality (Table 1). Among the ACS patients, 29 (61.7%) presented with acute myocardial infarction and 18 (38.3%) with unstable angina. The age distribution was comparable between groups, with mean ages of 52.86 ± 10.86 years for AMI patients, 50.89 ± 10.86 years for unstable angina patients, and 49.80 ± 10.50 years for controls (*p* = 0.531; Table 1).

**Figure 1:**
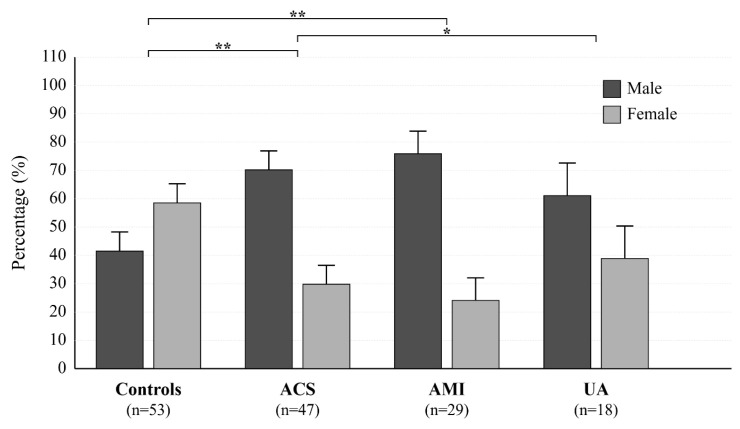
Gender distribution in acute coronary syndrome and control groups. Comparison of gender distribution between healthy controls (*n* = 53) and patients with acute coronary syndrome (ACS; *n* = 47), with subgroup analysis of acute myocardial infarction (AMI; *n* = 29) and unstable angina (UA; *n* = 18). Values represent percentage of male (dark gray) and female (light gray) subjects within each group. A significant male predominance was observed in ACS patients (70.2%) compared with controls (41.5%; *p* = 0.003). When ACS was subdivided into AMI (75.9% male) and UA (61.1% male) subgroups, the overall comparison remained significant (*p* = 0.009). Data are presented as percentages with error bars representing standard error of the mean. Statistical comparisons between groups were performed using chi-square test. *p*-values are reported in the text and tables.* denotes *p* < 0.05; ** denotes *p* < 0.01.

A significant male predominance was observed among ACS patients compared with controls, as illustrated in Figure 1 and detailed in Table 1. ACS patients showed significantly higher male representation (33/47; 70.2%) compared with controls (22/53; 41.5%; *p* = 0.003). This gender disparity was most pronounced in the AMI subgroup, where males comprised 75.9% of patients, followed by unstable angina, with 61.1% male patients, contrasting sharply with the control group, where males represented only 41.5% of participants (Figure 1).

### 3.2. Traditional Cardiovascular Risk Factors

The distribution of traditional cardiovascular risk factors between ACS patients and controls is presented in Table 2. Diabetes mellitus was more prevalent among ACS patients (61.7%) than controls (28.3%; OR = 4.08, 95% CI: 1.76–9.45; *p* = 0.001; Table 2).

Current or former smoking was highly prevalent in both groups (89.4% of ACS patients vs. 83.0% of controls), and binary smoking status did not differ significantly between groups (OR = 1.72, 95% CI: 0.54–5.44, and *p* = 0.356; Table 2), reflecting limited variability due to the high baseline prevalence in both populations. However, when smoking exposure was quantified using the smoking index (cigarettes/day × years), ACS patients demonstrated significantly greater cumulative smoking burden compared with controls (741.4 vs. 378.1 cigarettes/day × years; *p* = 0.036; Table 1). Given that cumulative exposure better captures the overall adverse physiological load from smoking, the dichotomous measure appears to be an inadequate indicator in populations with near-universal smoking prevalence.

Dyslipidemia was present in 55.3% of ACS patients versus 37.7% of controls (OR = 2.04, 95% CI: 0.92–4.60, and *p* = 0.080; Table 2), though not reaching statistical significance (*p* = 0.080). The observed effect size suggests dyslipidemia may contribute to ACS risk, though this study may have been underpowered to detect this relationship definitively given the sample size constraints.

### 3.3. Inflammatory Response and CRP

**Figure 2:**
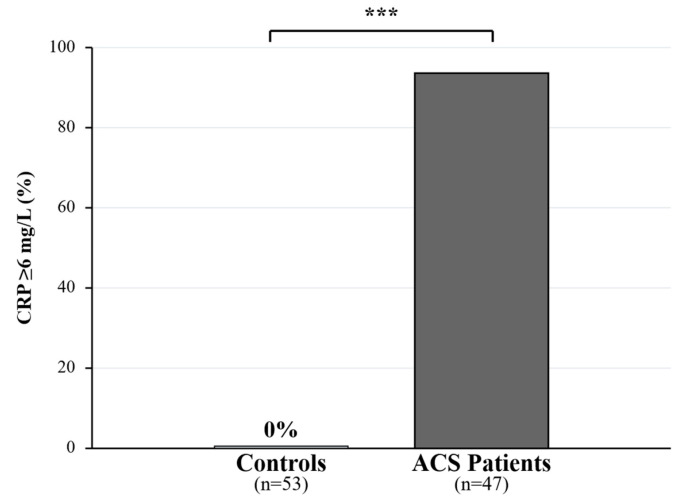
Association of CRP elevation with acute coronary syndrome. Percentage of participants with elevated CRP (≥6 mg/L) in ACS patients versus controls. Elevated CRP was observed in 93.6% of ACS patients compared with 0% of controls (*p* < 0.001). The Y-axis represents the proportion (%) of subjects with CRP ≥ 6 mg/L within each group. Data are presented as percentages with error bars representing standard error of the proportion. Statistical significance between groups was assessed using the chi-square test; *** denotes *p* < 0.001.

CRP elevation (≥6 mg/L), a non-specific marker of acute inflammation, was observed in 44 of 47 ACS patients (93.6%) compared with none of the 53 control subjects (0%; *p* < 0.001; Table 1 and Figure 2). This finding is consistent with the expected acute-phase response accompanying myocardial injury and does not, by itself, implicate a specific infectious etiology.

### 3.4. Chlamydia pneumoniae Serological Results

The serological findings for *C. pneumoniae* are detailed in Table 3. No participants in either group demonstrated IgM seropositivity for *C. pneumoniae*, indicating absence of acute chlamydial infection in all study subjects. IgG seropositivity, indicative of chronic or past infection, showed significant differences between groups. *C. pneumoniae* IgG seropositivity was markedly higher among ACS patients (39/47; 83.0%) compared with controls (32/53, 60.4%; OR = 3.20, 95% CI: 1.23–8.30, and *p* = 0.017; Table 3). Subgroup analysis revealed consistent patterns across ACS subtypes, with seropositivity rates of 86.2% in AMI patients and 77.8% in unstable angina patients, both elevated compared with the 60.4% rate observed in controls.

### 3.5. Multivariate Analysis of Risk Factor Associations

**Figure 3:**
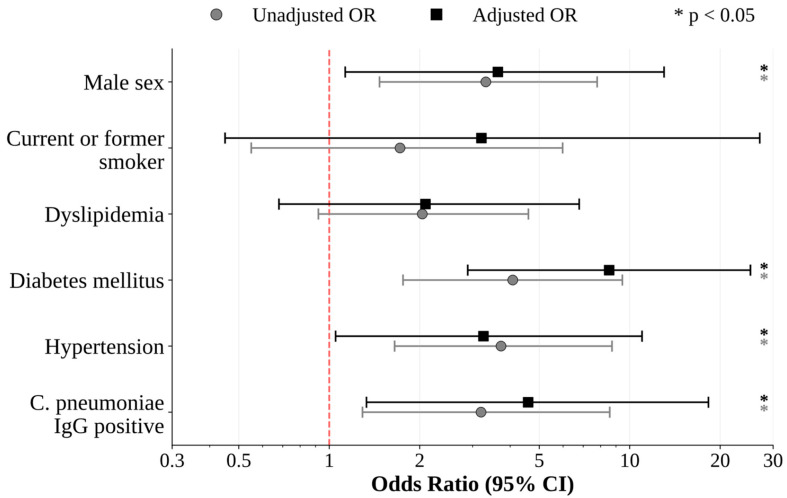
Multivariate analysis of risk factor associations with acute coronary syndromes. Forest plot showing odds ratios (ORs) with 95% confidence intervals (CIs) for risk factors associated with ACS. Gray circles represent unadjusted ORs; black squares represent adjusted ORs from multivariate logistic regression controlling for all variables shown. Horizontal lines indicate 95% CIs. The vertical red dashed line at OR = 1 represents no association. Statistical significance was defined as *p* < 0.05; actual *p*-values are reported in Table 4. After adjustment, diabetes mellitus (OR = 8.54), male sex (OR = 3.64), and hypertension (OR = 3.26) remained associated with ACS. *C. pneumoniae* IgG positivity (OR = 4.59) showed a suggestive adjusted association that did not meet the prespecified multivariate threshold of *p* < 0.01 (*p* = 0.021). X-axis is on a logarithmic scale. OR, odds ratio; CI, confidence interval; ACS, acute coronary syndrome.

The multivariate logistic regression analysis results are presented in Table 4 and visualized in the forest plot (Figure 3). All traditional cardiovascular risk factors demonstrated significant crude associations with ACS (male sex: OR = 3.32, smoking: OR = 1.72 (NS), hypertension: OR = 3.73, and diabetes: OR = 4.08; Table 4), and *C. pneumoniae* IgG positivity retained a suggestive signal in the multivariate model adjusted for smoking, hypertension, diabetes mellitus, and dyslipidemia (adjusted OR: 4.59, 95% CI: 1.33–18.28, and *p* = 0.021; Table 4). This estimate did not reach our prespecified multivariate threshold of *p* < 0.01, and the confidence interval was wide, reflecting the modest events-per-variable ratio. The result is best interpreted as suggestive of, rather than confirming, an independent association between *C. pneumoniae* seropositivity and acute coronary syndromes, beyond the contribution of conventional cardiovascular risk factors, although the case–control design precludes causal inference.

### 3.6. Clinical Correlates of *C. pneumoniae* IgG Seropositivity Within the ACS Cohort

**Figure 4:**
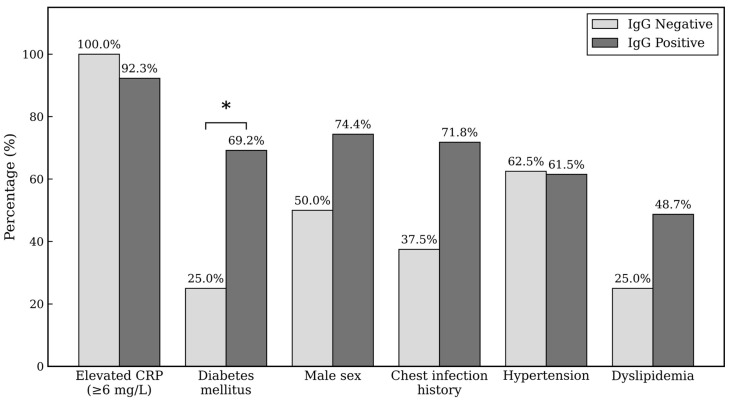
Association Between *C. pneumoniae* IgG Seropositivity and Clinical Variables in ACS Patients. Bar chart comparing cardiovascular risk factors stratified by *C. pneumoniae* IgG serostatus in ACS patients (*n* = 47). Light gray bars: IgG-negative (*n* = 8); dark gray bars: IgG-positive (*n* = 39). Data are presented as percentages within each serological group. Elevated CRP (≥6 mg/L) was present in 100% of seronegative patients versus 92.3% of seropositive patients (Fisher’s exact *p* = 1.000). Diabetes mellitus prevalence was numerically higher in seropositive patients (69.2% vs. 25.0%, Fisher’s exact *p* = 0.041, borderline). Other clinical variables showed no statistically significant associations with *C. pneumoniae* serostatus: male sex (74.4% vs. 50.0%, *p* = 0.215), chest infection history (71.8% vs. 37.5%, *p* = 0.101), hypertension (61.5% vs. 62.5%, *p* = 1.000), and dyslipidemia (48.7% vs. 25.0%, *p* = 0.269). All *p*-values were derived from Fisher’s exact test. Actual *p* values are reported in the text and Table 5. Abbreviations: CRP, C-reactive protein; IgG, immunoglobulin G; ACS, acute coronary syndromes. * denotes statistical significance at *p* < 0.05 (Fisher’s exact test).

Within the ACS patient cohort, we examined the relationship between *C. pneumoniae* IgG seropositivity and various clinical characteristics, as detailed in Table 5 and illustrated in Figure 4. CRP elevation was near-universal in both serogroups. Of the 39 *C. pneumoniae*-seropositive ACS patients, 36 (92.3%) had elevated CRP levels, compared with eight of eight (100%) seronegative patients; in Fisher’s exact test, this difference was not statistically significant (*p* = 1.000; Table 5). Within the ACS cohort, IgG seropositivity did not predict CRP elevation.

Other clinical associations within the ACS group showed varying degrees of correlation with *C. pneumoniae* seropositivity (Table 5). Diabetes mellitus prevalence was numerically higher among seropositive patients (69.2%) than seronegative patients (25.0%; Fisher’s exact *p* = 0.041), a borderline difference. Male gender was present in 74.4% of seropositive patients versus 50.0% of seronegative patients (Fisher’s exact *p* = 0.215).

Hypertension was observed in 61.5% of seropositive versus 62.5% of seronegative patients (Fisher’s exact *p* = 1.000), while dyslipidemia affected 48.7% of seropositive versus 25.0% of seronegative patients (Fisher’s exact *p* = 0.269). A history of chest infections was more prevalent among *C. pneumoniae*-seropositive patients (71.8%) compared with seronegative patients (37.5%), though this difference was not statistically significant (Fisher’s exact *p* = 0.101).

### 3.7. CRP Elevation and Associated Clinical Characteristics

The association between CRP elevation and clinical characteristics within the ACS population is examined in Table 6. Among the 44 ACS patients with elevated CRP, *C. pneumoniae* IgG seropositivity was present in 36 patients (81.8%), while all three patients with normal CRP levels were also seropositive for *C. pneumoniae*. In Fisher’s exact test, this difference was not statistically significant (*p* = 1.000; Table 6), reflecting the small number of patients with normal CRP (*n* = 3) and the near-universal CRP elevation across both serogroups.

The clinical profile of patients with elevated CRP showed distinct patterns compared with those with normal CRP levels (Table 6). Diabetes mellitus prevalence was numerically higher among patients with elevated CRP (65.9%) compared with those with normal CRP levels (0%; Fisher’s exact *p* = 0.050, borderline). Hypertension prevalence did not differ significantly between patients with elevated CRP (63.6%) and those with normal CRP (33.3%), on Fisher’s exact test (*p* = 0.549). With only three patients in the CRP-normal subgroup, these comparisons are markedly underpowered and should be interpreted as descriptive.

## 4. Discussion

Investigation of the link between chronic *C. pneumoniae* infection and atherosclerotic cardiovascular disease dates to Saikku et al.’s seminal seroepidemiological observation in 1988 [41]. We found significantly elevated *C. pneumoniae* IgG seropositivity in ACS patients compared with controls in unadjusted analysis (OR: 3.20; 95% CI: 1.23–8.30; *p* = 0.017). After multivariable adjustment, the signal remained suggestive (adjusted OR: 4.59; 95% CI: 1.33–18.28; *p* = 0.021) but did not meet our prespecified multivariate threshold of *p* < 0.01 and, given the wide confidence interval, is best regarded as exploratory rather than confirmatory. The finding aligns with prior work yet leaves causality, mechanism, and therapeutic implications open. CRP elevation was present in 93.6% of ACS patients and in none of the controls. The pattern reflects the non-specific acute-phase response to myocardial injury rather than infection-specific inflammation. Enrollment of 100 participants (47 cases; 53 controls) exceeded the calculated requirement of 42 per group. The target was to detect a 30% difference in seropositivity at 80% power and α = 0.05. Power was adequate against the planned effect size. The observed 22.6% difference (83.0% vs. 60.4%) fell below this threshold. The primary comparison was, therefore, marginally underpowered, and subgroup analyses or formal interaction tests were constrained.

Several established cardiovascular risk factors were independently associated with ACS in our cohort. Diabetes mellitus carried a strong association (OR = 4.08; *p* = 0.001) that strengthened on adjustment (adjusted OR = 8.54; *p* < 0.001). The signal is in keeping with its known effects on endothelial function, accelerated atherogenesis, and the prothrombotic state. Glycemic injury to the vascular wall remains a plausible mediating pathway. Hypertension affected 61.7% of cases versus 30.2% of controls. It remained an independent predictor (OR = 3.73 (*p* = 0.001); adjusted OR = 3.26 (*p* = 0.046)). The magnitude of effect is consistent with the contribution of pressure-related endothelial damage to plaque instability.

Dyslipidemia affected 55.3% of ACS patients and 37.7% of controls. The unadjusted OR was 2.04 (95% CI: 0.92–4.60; *p* = 0.080). The point estimate did not reach conventional significance. The roughly twofold increase in odds is plausibly clinically relevant. Our study may have been underpowered for this comparison. The high prevalence in both groups likely reflects regional dietary patterns and the heavy burden of metabolic syndrome in this Egyptian population. Such background can reduce the discriminatory power of dyslipidemia as a risk factor in our cohort. Dyslipidemia remains a recognized driver of atherogenesis, and its near-equal distribution between cases and controls likely diluted rather than abolished the biological effect.

Binary smoking status did not differ between groups (OR = 1.72; *p* = 0.356). High baseline prevalence in both arms constrained discrimination (89.4% in cases versus 83.1% in controls). The smoking index, defined as cigarettes per day multiplied by years smoked, captures the cumulative dose. The index was substantially higher in ACS patients (741.4 versus 378.1; *p* = 0.036). Cumulative exposure captures physiological load that the binary measure cannot. In populations with near-universal smoking, quantitative exposure metrics outperform categorical classification for risk stratification. Pairing the two measures here exposed dose–response information that dichotomous coding alone would have obscured. Within smoking-prevalent cohorts, this dual approach is a methodological strength rather than a redundancy. The smoking index has the further advantage of preserving an ordinal gradient that survives saturation of the dichotomous indicator.

*C. pneumoniae* is an obligate intracellular bacterium that causes about 10% of community-acquired pneumonia. Adult seroprevalence reaches 50–70% and rises with age [42]. A biphasic developmental cycle alternates between infectious elementary bodies and metabolically active reticulate bodies. Transmission and intracellular persistence both depend on this switching [43]. Chronic infection is established by interference with host apoptotic pathways and by evasion of lysosomal fusion in respiratory and vascular cells [43].

From the respiratory tract, *C. pneumoniae* disseminates within infected monocytes [44,45]. The vascular wall is the eventual site. There, the organism infects endothelial cells, smooth muscle cells, and macrophages [35,46,47]. Local inflammation begins with NF-κB activation. Induction of adhesion molecules and release of pro-inflammatory cytokines follow [48]. Oxidative stress with LDL oxidation and foam cell formation has also been described [45,49]. Molecular mimicry between chlamydial and human heat shock protein 60 can trigger autoimmune vascular injury [50,51,52,53,54].

When stratified by IgG status, CRP elevation reached 92.3% in seropositive and 100% in seronegative ACS patients. Upon re-analysis with Fisher’s exact test, this difference was not statistically significant (*p* = 1.000; Table 5 and Table 6). The inverse stratification of IgG serostatus by CRP level showed 81.8% IgG positivity among CRP-elevated patients and 100% IgG positivity among the three CRP-normal patients, again, with no significant difference (Fisher’s exact *p* = 1.000). The near-universal CRP elevation, irrespective of *C. pneumoniae* serological status, undermines a proposed mechanistic link between chlamydial infection and CRP elevation in the acute setting. In this context, CRP reflects the acute vascular event itself rather than underlying chronic infection. Elevation in 93.6% of ACS patients matches the expected non-specific acute-phase response to myocardial injury. The pattern should not be read as evidence of infection-driven inflammation. The likely sources are myocardial necrosis, plaque disruption, and the resulting systemic inflammatory cascade rather than chronic *C. pneumoniae* infection.

Investigators have linked CRP elevation to *C. pneumoniae* seropositivity in coronary disease across several studies. Mayr et al. [55] reported an odds ratio of 4.2 for carotid atherosclerosis in seropositive subjects with elevated CRP. The OR rose to 6.3 in those with chronic respiratory infection. The meta-analysis by Filardo et al. [56] showed higher CRP, IL-6, and fibrinogen in *C. pneumoniae* IgA-seropositive atherosclerotic patients than in seronegative patients. Johnston et al. [57] recovered viable *C. pneumoniae* from 38% of carotid plaques. Infected plaques had a median CRP of 8 mg/L versus undetectable in non-infected plaques. Causality remains unresolved. CRP could reflect chlamydial inflammation. Alternatively, individuals with heightened inflammatory tone may be more susceptible to both chronic infection and atherosclerosis, a reverse causation pattern. Song et al. [58] argued that CRP and other inflammatory markers contribute more to ACS pathogenesis than *C. pneumoniae* infection itself. IgM antibodies were absent throughout our cohort, in line with reports of high seroprevalence without acute IgM responses [59]. The pattern indicates chronic infection and suggests that any contribution to ACS operates through chronic mechanisms rather than acute infection-triggered plaque rupture.

Whether *C. pneumoniae* plays a direct pathogenic role in atherosclerosis remains unresolved. Detection rates have ranged from 34 to 71% in atheromatous tissue versus 5–9% in normal arteries across published series [60,61,62]. Critical appraisals point to substantial obstacles in establishing causality [32]. The innocent bystander hypothesis holds that *C. pneumoniae* preferentially colonizes pre-existing atherosclerotic lesions. The argument rests on its detection in other pathological tissues and its infrequent presence in healthy non-cardiovascular tissue [60]. Some authors invoke contributory mechanisms involving inflammation and cellular dysfunction [35]. Others emphasize the lack of conclusive evidence for a causal role [31,62]. Antibiotic intervention trials have given mixed results. The question remains open [33,42]. Seroepidemiological results are technique-dependent. PCR estimates vary widely, and detection often fails once assay specificity is tightened [32,63]. Detection of *C. pneumoniae* in non-cardiovascular tissues fits opportunistic colonization [60]. Available evidence supports a contributory rather than primary pathogenic role.

Large randomized trials of antibiotic therapy for secondary coronary prevention have shown no benefit. The pattern challenges the infection hypothesis of atherosclerosis. WIZARD (*n* = 7747) found no reduction in cardiovascular events with azithromycin after prior myocardial infarction [64]. Zahn et al. found no mortality benefit from roxithromycin among 872 acute myocardial infarction patients [65]. A meta-analysis of nine trials (*n* = 11,015) showed no significant reduction in recurrent cardiac events or mortality with macrolide therapy [66]. CLARICOR (*n* = 4373) returned excess cardiovascular mortality on clarithromycin versus placebo [67]. PROVE-IT TIMI 22 showed that 80 mg of intensive atorvastatin lowered cardiovascular events relative to 40 mg of pravastatin [68]. Its factorial antibiotic arm (*n* = 4162) found no benefit of gatifloxacin, even in patients with elevated *C. pneumoniae* titers [69]. Anderson and Muhlestein concluded that standard antibiotic regimens fail in secondary cardiovascular prevention despite continuing observational links between infection and atherothrombosis [70]. The aggregate signal across these data is no protection. Negative trials do not, however, disprove the infectious hypothesis [71]. Treatment of established disease may simply be too late to alter atherogenesis. *C. pneumoniae* forms persistent, metabolically inactive bodies that confer relative antibiotic resistance. Viable organisms may persist in vascular tissue beyond apparent clearance from blood and respiratory compartments. Infection can also trigger self-perpetuating inflammatory cascades that outlast pathogen eradication [42,72]. A pathogen burden hypothesis posits synergistic contribution from multiple organisms [73]. Single-pathogen targeting would then be inadequate. Trial failures have nevertheless dampened research interest in infectious etiologies of atherosclerosis.

Serological testing for *C. pneumoniae* carries methodological limits that constrain its use in cardiovascular research. Microimmunofluorescence (MIF), the reference assay, shows substantial interlaboratory variation. Overall agreement between laboratories is only about 80%. Coefficients of variation exceed acceptable bounds [74]. The variability traces to lack of standardization in assay conditions and interpretation [75]. Serological markers correlate poorly with actual vascular infection. MIF reached only 21% sensitivity and a 37% positive predictive value against PCR detection of *C. pneumoniae* in coronary artery segments [76]. ELISA-based testing in our study sits within these broader constraints. Commercial ELISA assays are more reproducible than MIF yet lack standardized cut-off points for seropositivity. Investigators apply arbitrary thresholds that vary across studies, fragmenting comparability across cohorts. Agreement is adequate only at low antibody titers [77]. Reliability falls in the very range where chronic infection markers would lie. We added CRP as a complementary inflammatory marker. Doing so adds biological plausibility. Among CRP-elevated ACS patients, 81.8% were *C. pneumoniae*-seropositive (Table 6). No controls had CRP elevation. The combination supports an inflammatory process but cannot resolve the underlying diagnostic limitation. CRP itself is non-specific. It could reflect chlamydial vascular inflammation or atherosclerotic processes that predispose to bacterial colonization. Technical limitations contribute directly to the conflicting findings that have characterized the field. Similar populations yield divergent conclusions based on serological methodology and marker selection alone. Validated cut-off points are absent. With antibody titers, a poor proxy for vascular bacterial burden, serological evidence for a cardiovascular role of *C. pneumoniae* remains inherently unreliable. Standardized, histologically validated diagnostic criteria are required. Until direct pathogen detection is validated and standardized, the causation–correlation dilemma will persist regardless of how many indirect markers are combined.

### Limitations

The case–control design precludes temporal inference. Whether infection preceded atherosclerosis or whether cardiovascular disease itself produced immune alterations favoring chronic infection cannot be determined here (reverse causality). Despite multivariate adjustment, residual confounding from unmeasured variables remains possible. Recruitment was from Alexandria, Egypt. Infectious disease patterns there differ from Western populations, which may limit generalizability. The modest sample (47 cases and 53 controls) reduces power for weaker associations and for risk factor interactions. Including six predictors in a multivariable logistic model with only 47 cases raises a concern for overfitting. Several adjusted odds ratios carry wide confidence intervals (diabetes mellitus: 2.89–25.22; *C. pneumoniae* IgG: 1.33–18.28). The breadth of those intervals limits the precision of the estimates. Hosmer–Lemeshow goodness-of-fit testing and other model diagnostics were not performed. The multivariable results should, therefore, be read as exploratory rather than confirmatory. Steyerberg et al. [78] and Harrell et al. [79] have emphasized that small datasets with low events-per-variable ratios produce biased coefficients and unstable estimates. We also used unconditional rather than conditional logistic regression. Conditional regression would have been the more appropriate choice for a matched case–control design. It accommodates matching variables that are not fully captured in the model, although age was included as a covariate. Sex was not a matching variable. The resulting imbalance (70.2% male in ACS versus 41.5% in controls) may have shifted associations for other variables. Bivariate analyses used a *p* < 0.05 threshold without correction for multiple comparisons, raising the risk of false-positive findings. Borderline associations should be read accordingly. Single time-point serology gives no information on chronicity or bacterial load. Antibody detection signals exposure, not active vascular infection. Socioeconomic factors, concurrent infections, and genetic polymorphisms were not measured. Each could shape susceptibility to infection and cardiovascular outcomes beyond the variables in our model.

With these caveats, our results replicate the reported association between chronic *C. pneumoniae* seropositivity and ACS, this time in an Egyptian population. They add to the seroepidemiological literature rather than offering new mechanistic insight. Statistical independence in a multivariable model does not equal biological causation. The observed association is equally consistent with an alternative reading. Patients with advanced atherosclerosis and heightened inflammatory tone may simply have higher seropositivity. This would mark cumulative exposure or immune activation rather than infection-triggered ACS. Large randomized antibiotic trials have not improved cardiovascular outcomes. Their record argues against a primary causal role. A parsimonious reading is that *C. pneumoniae* seropositivity modulates the inflammatory milieu of atherosclerosis without driving it. The relevant test going forward is whether any infection-associated inflammatory pathway offers a tractable therapeutic target. Approaches beyond standard antibiotics deserve formal evaluation in adequately powered trials. Anti-inflammatory strategies and combined targeting of plausible co-pathogens are the leading candidates.

## 5. Conclusions

Within our 100-participant case–control sample (47 ACS; 53 controls), *C. pneumoniae* IgG seropositivity was associated with ACS in unadjusted analysis (OR: 3.20; 95% CI: 1.23–8.30; *p* = 0.017). After multivariable adjustment, the signal persisted (adjusted OR: 4.59; 95% CI: 1.33–18.28; *p* = 0.021) but did not reach our prespecified threshold of *p* < 0.01 and is best regarded as suggestive rather than confirmed, given the modest sample size and wide confidence interval. IgG was present in the absence of IgM, a pattern consistent with prior exposure or chronic infection. Seropositivity does not, however, distinguish past immunological contact from active pathogenic involvement of the vascular wall. This serological constraint precludes firm causal inference. CRP elevation reached 93.6% in ACS patients against 0% in controls. Such a CRP signal is non-specific in this clinical context. The most parsimonious reading is the acute-phase response to myocardial injury rather than infection-driven inflammation. Reverse causality and residual confounding remain open under the observational design. Antibiotic trials for secondary cardiovascular prevention have not improved outcomes. WIZARD (*n* = 7747), CLARICOR (*n* = 4373), and the PROVE-IT TIMI 22 antibiotic arm (*n* = 4162) all returned null or unfavorable results. The aggregate negative-trial evidence is incompatible with a primary causal role for *C. pneumoniae* in ACS. Any contribution by the organism is more plausibly modulation of the inflammatory milieu than primary causation. *C. pneumoniae* seropositivity is best read as a marker of increased cardiovascular risk rather than a validated therapeutic target. Prospective studies pairing direct pathogen detection with mechanistic validation are needed before infection-targeted interventions can be advanced for cardiovascular prevention.

## Figures and Tables

**Table 1:** Baseline characteristics of study participants.

Characteristic	ACS Patients (*n* = 47)	Controls (*n* = 53)	*p*-Value
Age (years)			
Mean ± SD	52.1 ± 10.4 ᵃ	49.8 ± 10.5	0.531
Range	32–75	33–71	
Male sex, no. (%)	33 (70.2)	22 (41.5)	0.003
ACS subtype, no. (%)			
Acute myocardial infarction	29 (61.7)	---	
Unstable angina	18 (38.3)	---	
Traditional cardiovascular risk factors, no. (%)			
Current or former smoking	42 (89.4)	44 (83.1)	0.034
Mean smoking index (cigarettes/day × years)	741.4	378.1	0.036
Hypertension ᵇ	29 (61.7)	16 (30.2)	0.003
Diabetes mellitus ^c^	29 (61.7)	15 (28.3)	0.001
Dyslipidemia ᵈ	26 (55.3)	20 (37.7)	0.080
Laboratory parameters			
Elevated CRP (≥6 mg/L), No. (%)	44 (93.6)	0 (0)	<0.001

Baseline demographic, clinical, and laboratory characteristics of acute coronary syndrome (ACS) patients and age- and locality-matched controls. Continuous variables are expressed as means ± standard deviation and compared with Student’s *t*-test; categorical variables are expressed as numbers (percentages) and compared using chi-square or Fisher’s exact test. Footnotes: ᵃ Weighted mean age across ACS subtypes: AMI: 52.86 ± 10.13 years; UA: 50.89 ± 10.86 years. ᵇ Hypertension was defined as systolic blood pressure ≥ 140 mmHg, diastolic blood pressure ≥ 90 mmHg, or antihypertensive medication use. ᶜ Diabetes mellitus was defined as fasting plasma glucose ≥ 126 mg/dL or antidiabetic medication use. ᵈ Dyslipidemia was defined as total cholesterol > 200 mg/dL, LDL ≥ 130 mg/dL, HDL < 40 mg/dL (men) or < 45 mg/dL (women), or triglycerides > 180 mg/dL. CRP was measured by latex agglutination test on admission.

**Table 2:** Association between traditional cardiovascular risk factors and acute coronary syndromes.

Risk Factor	ACS Patients (*n* = 47)	Controls (*n* = 53)	Odds Ratio (95% CI)	*p*-Value
Smoking status				
Never smoker	5 (10.6)	9 (17.0)	1.00 (Reference)	
Current or former smoker	42 (89.4)	44 (83.0)	1.72 (0.54 to 5.44)	0.356
Hypertension				
Absent	18 (38.3)	37 (69.8)	1.00 (Reference)	
Present	29 (61.7)	16 (30.2)	3.73 (1.66 to 8.39)	0.001
Diabetes mellitus				
Absent	18 (38.3)	38 (71.7)	1.00 (Reference)	
Present	29 (61.7)	15 (28.3)	4.08 (1.76 to 9.45)	0.001
Dyslipidemia				
Absent	21 (44.7)	33 (62.3)	1.00 (reference)	
Present	26 (55.3)	20 (37.7)	2.04 (0.92 to 4.60)	0.080

Logistic regression analysis of cardiovascular risk factors associated with ACS. Data are presented as numbers (percentages). Odds ratios (ORs) and 95% confidence intervals (CIs) are reported relative to the reference categories. Statistical significance was assessed using logistic regression analysis.

**Table 3:** *Chlamydia pneumoniae* serological results by study group. Serological detection of *Chlamydia pneumoniae* antibodies by enzyme-linked immunosorbent assay (ELISA). Data are presented as numbers (percentages). IgM indicates recent/acute infection; IgG indicates past exposure or chronic infection. The odds ratio and *p*-value are for the comparison of IgG seropositivity between the total ACS group and controls. Statistical significance was assessed using chi-square test.

Serological Marker	Total ACS (*n* = 47)	AMI (*n* = 29)	Unstable Angina (*n* = 18)	Controls (*n* = 53)	*p*-Value
***C. pneumoniae*** **IgM**					
Negative	47 (100)	29 (100)	18 (100)	53 (100)	
Positive	0 (0)	0 (0)	0 (0)	0 (0)	
***C. pneumoniae*** **IgG**					
Negative	8 (17.0)	4 (13.8)	4 (22.2)	21 (39.6)	
Positive	39 (83.0)	25 (86.2)	14 (77.8)	32 (60.4)	
**Odds ratio for IgG positivity (95% CI)**	3.20 (1.23–8.30)	—	—	Reference	0.017

**Table 4:** Univariate and multivariate logistic regression with calculated OR (95% CI).

Variable		Controls, *n* = 53	Cases, *n* = 47	Unadjusted OR (95% CI)	Adjusted OR (95% CI)
Sex	Female	31 (58.5)	14 (29.8)	---	---
	Male	22 (41.5)	33 (70.2)	3.32 (1.47–7.79, *p* = 0.005 *)	3.64 (1.13–13.01, *p* = 0.036 *)
Current or former smoker	No	9 (17.0)	5 (10.6)	---	---
	Yes	44 (83.0)	42 (89.4)	1.72 (0.54–5.44, *p* = 0.356)	3.21 (0.45–27.08, *p* = 0.262)
Dyslipidemia	No	33 (62.3)	21 (44.7)	---	---
	Yes	20 (37.7)	26 (55.3)	2.04 (0.92–4.60, *p* = 0.080)	2.09 (0.68–6.79, *p* = 0.203)
Diabetes mellitus	No	38 (71.7)	18 (38.3)	---	---
	Yes	15 (28.3)	29 (61.7)	4.08 (1.76–9.45, p = 0.001 *)	8.54 (2.89–25.22, p < 0.001 *)
Hypertension	No	37 (69.8)	18 (38.3)	---	---
	Yes	16 (30.2)	29 (61.7)	3.73 (1.66–8.39, *p* = 0.001 *)	3.26 (1.05–10.99, *p* = 0.046 *)
*C. pneumoniae* IgG positive	No	21 (39.6)	8 (17.0)	---	---
	Yes	32 (60.4)	39 (83.0)	3.20 (1.23–8.30, *p* = 0.017 *)	4.59 (1.33–18.28, *p* = 0.021 *)

Logistic regression analysis of risk factors associated with acute coronary syndromes. Data are presented as numbers (percentages). Unadjusted odds ratios (ORs) with 95% confidence intervals (CIs) represent crude associations. Adjusted ORs are derived from a multivariate model controlling for sex, smoking, dyslipidemia, diabetes mellitus, hypertension, and *C. pneumoniae* IgG seropositivity. Reference categories (indicated by “---”) are female sex, non-smoker, and absence of each risk factor. After multivariate adjustment, diabetes mellitus (OR = 8.54), male sex (OR = 3.64), and hypertension (OR = 3.26) remained associated with ACS. *C. pneumoniae* IgG positivity (OR = 4.59) showed a suggestive adjusted association that did not meet the prespecified multivariate threshold of *p* < 0.01 (*p* = 0.021). OR: odds ratio; CI: confidence interval. Statistical significance was defined as *p* < 0.05; actual *p*-values are reported for each variable. * Denotes statistical significance at *p* < 0.05.

**Table 5:** Association between *C. pneumoniae* seropositivity and clinical variables in ACS patients.

Variable	IgG Negative (*n* = 8)	IgG Positive (*n* = 39)	Total (*n* = 47)	*p*-Value
Demographics				
Male sex, no. (%)	4 (50.0)	29 (74.4)	33 (70.2)	0.215
Cardiovascular risk factors, no. (%)				
Hypertension	5 (62.5)	24 (61.5)	29 (61.7)	1.000
Diabetes mellitus	2 (25.0)	27 (69.2)	29 (61.7)	0.041
Dyslipidemia	2 (25.0)	19 (48.7)	21 (44.7)	0.269
Smoking history	7 (87.5)	35 (89.7)	42 (89.4)	1.000
Clinical history, no. (%)				
Chest infection history	3 (37.5)	28 (71.8)	31 (66.0)	0.101
Inflammatory marker, no. (%)				
Elevated CRP (≥6 mg/L)	8 (100.0)	36 (92.3)	44 (93.6)	1.000

Comparison of clinical characteristics stratified by *C. pneumoniae* IgG serostatus within the ACS patient group (*n* = 47). Data are presented as numbers (percentages). All *p*-values were recomputed using Fisher’s exact test on the cell counts as reported, given small expected cell counts in several cross-tabulations. Statistical significance was defined as *p* < 0.05. Note: The diabetes mellitus prevalence in this IgG-stratified analysis (*n* = 29; 61.7%) corresponds to the overall cohort total (Table 1). Diabetes prevalence was higher among IgG-seropositive patients (69.2% vs. 25.0%), with a borderline difference in Fisher’s exact test (*p* = 0.041).

**Table 6:** CRP elevation and associated clinical characteristics in ACS patients.

Variable	CRP Normal (*n* = 3)	CRP Elevated (*n* = 44)	Total (*n* = 47)	*p*-Value
*C. pneumoniae* serology, no. (%)				
IgG negative	0 (0.0)	8 (18.2)	8 (17.0)	1.000
IgG positive	3 (100.0)	36 (81.8)	39 (83.0)	
Demographics, no. (%)				
Male sex	2 (66.7)	31 (70.5)	33 (70.2)	1.000
Risk factors, no. (%)				
Hypertension	1 (33.3)	28 (63.6)	29 (61.7)	0.549
Diabetes mellitus	0 (0.0)	29 (65.9)	29 (61.7)	0.050
Dyslipidemia	2 (66.7)	24 (54.5)	26 (55.3)	1.000
Smoking history	1 (33.3)	41 (93.2)	42 (89.4)	0.027

Association between CRP elevation (≥6 mg/L) and clinical characteristics within the ACS patient group (*n* = 47). Data are presented as numbers (percentages). All *p*-values were recomputed using Fisher’s exact test on the cell counts as reported, given small expected cell counts in several cross-tabulations (*n* = 3 in the CRP-normal subgroup). Statistical significance was defined as *p* < 0.05.

## Data Availability

The data supporting the findings of this study are available from the corresponding author upon reasonable request.

## Abbreviations

The following abbreviations are used in this manuscript:

**Abbreviation**	**Definition**
ACS	Acute Coronary Syndrome
AMI	Acute Myocardial Infarction
CI	Confidence Interval
CK-MB	Creatine Kinase Myocardial Band
CRP	C-Reactive Protein
cHSP60	Chlamydial Heat Shock Protein 60
DNA	Deoxyribonucleic Acid
ELISA	Enzyme-Linked Immunosorbent Assay
HDL	High-Density Lipoprotein
HSP60	Heat Shock Protein 60
ICAM-1	Intercellular Adhesion Molecule-1
IL-1β	Interleukin-1 Beta
IL-6	Interleukin-6
IL-8	Interleukin-8
LDH	Lactate Dehydrogenase
LDL	Low-Density Lipoprotein
MAPK	Mitogen-Activated Protein Kinase
MIF	Microimmunofluorescence
NF-κB	Nuclear Factor Kappa B
OR	Odds Ratio
PCR	Polymerase Chain Reaction
SD	Standard Deviation
SMuRFs	Standard Modifiable Risk Factors
UA	Unstable Angina
VCAM-1	Vascular Cell Adhesion Molecule-1
